# Evaluating the Bioenergetics Health Index Ratio in Leigh Syndrome Fibroblasts to Understand Disease Severity

**DOI:** 10.3390/ijms221910344

**Published:** 2021-09-26

**Authors:** Ajibola B. Bakare, Joseph Dean, Qun Chen, Vedant Thorat, Yimin Huang, Thomas LaFramboise, Edward J. Lesnefsky, Shilpa Iyer

**Affiliations:** 1 Department of Biological Sciences, J. William Fulbright College of Arts and Sciences, University of Arkansas, Fayetteville, AR 72701, USA; abbakare@uark.edu; 2 Cell and Molecular Biology Program, University of Arkansas, Fayetteville, AR 72701, USA; 3 Cardiology Section Medical Service, McGuire Veterans Affairs Medical Center, Richmond, VA 23284, USA; Jdean1299@gmail.com (J.D.); Edward.Lesnefsky@va.gov (E.J.L.); 4 Pauley Heart Center, Division of Cardiology, Department of Internal Medicine, Virginia Commonwealth University, Richmond, VA 23284, USA; qun.chen@vcuhealth.org; 5 Department of Genetics and Genome Sciences, Case Western Reserve University School of Medicine, Cleveland, OH 44106, USA; vst14@case.edu (V.T.); yxh849@case.edu (Y.H.); txl80@case.edu (T.L.); 6 Department of Biochemistry and Molecular Biology, Virginia Commonwealth University, Richmond, VA 23219, USA

**Keywords:** mitochondrial disorders, leigh syndrome, glycolysis, mitochondrial respiration, bioenergetics health index

## Abstract

Several pediatric mitochondrial disorders, including Leigh syndrome (LS), impact mitochondrial (mt) genetics, development, and metabolism, leading to complex pathologies and energy failure. The extent to which pathogenic mtDNA variants regulate disease severity in LS is currently not well understood. To better understand this relationship, we computed a glycolytic bioenergetics health index (BHI) for measuring mitochondrial dysfunction in LS patient fibroblast cells harboring varying percentages of pathogenic mutant mtDNA (*T8993G*, *T9185C*) exhibiting deficiency in complex V or complex I (*T10158C*, *T12706C*). A high percentage (>90%) of pathogenic mtDNA in cells affecting complex V and a low percentage (<39%) of pathogenic mtDNA in cells affecting complex I was quantified. Levels of defective enzyme activities of the electron transport chain correlated with the percentage of pathogenic mtDNA. Subsequent bioenergetics assays showed cell lines relied on both OXPHOS and glycolysis for meeting energy requirements. Results suggest that whereas the precise mechanism of LS has not been elucidated, a multi-pronged approach taking into consideration the specific pathogenic mtDNA variant, glycolytic BHI, and the composite BHI (average ratio of oxphos to glycolysis) can aid in better understanding the factors influencing disease severity in LS.

## 1. Introduction

Mitochondrial (mt) disorders represent a large group of severe genetic disorders mainly impacting organ systems with high energy requirements [1,2]. These disorders are clinically complex, often fatal, and occur at an estimated ratio of 1 in 5000 live births [3,4]. Although much progress has been made since the discovery of pathogenic mtDNA, we still do not understand whether pathogenic mtDNA directly or indirectly influences clinical severity. Previous studies indicate that the unorthodox genetics of a pathogenic mtDNA variant can influence clinical pathologies [5] because each mitochondrion contains hundreds of mtDNA existing as mixtures of wild type and pathogenic mtDNA molecules within a single cell termed as heteroplasmy. Heteroplasmy at both the cellular and tissue level is capable of shifting the fraction of mutant mtDNA that is present within the daughter cells by replicative segregation [6,7,8].

In addition, each cell contains a varying number of mitochondria based on the energy requirement of the specific tissue. Key processes including adenosine triphosphate (ATP) synthesis [9], tricarboxylic acid cycle (TCA), and fatty acid beta-oxidation [10] provide cellular ATP by transporting electrons generated from the oxidation of TCA cycle intermediates through the four electron transport chain (ETC) complexes coupled to the vectoral transport of protons to generate the proton motive force used by complex V to synthesize ATP [9]. During the electron transfer to molecular oxygen, reactive oxygen species (ROS) are generated by leakage of electrons in complex I and III causing oxidative stress to cells [9,10,11]. ETC defects occurring from mtDNA mutations compromise mitochondrial membrane potential and ATP synthesis via oxidative phosphorylation, and interruption of this pathway renders cells and tissues vulnerable under disease and oxidative stress conditions [12,13].

In this study, Leigh Syndrome (LS), a classic mitochondrial disorder, was selected to better understand the relationship between disease severity and its associated pathogenic mtDNA variants, heteroplasmy, and biochemical phenotypes reported in LS [2,14,15,16,17,18,19,20,21]. To date, the mechanisms causing LS are not well understood. Studies have reported LS symptoms as symmetrical necrotic lesions in the brain stem, basal ganglia, and thalamus [22]. Other studies have reported lactic acidosis, psychomotor retardation, failure to thrive, vomiting, seizures, respiratory failure, and ultimately death, thus restricting treatment options. Many LS patients have also shown elevated lactate in blood and cerebral spinal fluid (CSF) [23,24,25]. Currently, ~100 genes have been identified as monogenic causes of LS [2,22,26,27].

Earlier studies demonstrated that a high percentage of pathogenic mtDNA causing complex V deficiency contributes to maternally inherited LS (MILS) [21]. Results pointed out that *T8993G* mutation in high abundance (>90%) in the *MTATP6* gene resulted in MILS and caused neurologic findings, including seizures, respiratory dysfunction, and rapid fatality [19]. Recent studies in LS patients exhibiting varying levels of heteroplasmy in pathogenic mtDNA variants in the *MTATP6* gene reported hyperventilation at the onset of the disease [28]. Seminal studies in samples containing pathogenic mtDNA variants causing complex I deficiency showed that clinical symptoms were present even at low levels of heteroplasmy, with heterogeneous clinical outcomes ranging from neonatal lactic acidosis [29], optic neuropathy to LS [30] in children.

To better comprehend the clinical presentation associated with the pathogenic mtDNA variants, heteroplasmy levels, and their biochemical defects in LS, we selected five pediatric patient fibroblast cell lines with varying clinical presentations, from mild myopathies to severe LS. These cell lines carried point mutations in their mtDNA at *T8993G*, *T9185C* in *MTATP6* gene causing complex V deficiency and *T10158C* in *MTND3* gene and *T12706C* in *MTND5* gene causing complex I deficiency. A commercially available fibroblast cell line (BJ-FB) was used as a healthy control line. In this study, we attempt to systematically connect pathogenic mtDNA variants and associated bioenergetic defects and propose a composite bioenergetic health index ratio as a sensitive marker for assessing patient health in young children suffering from Leigh syndrome.

## 2. Results

### 2.1. Clinical Information and Fibroblast Cell Line Characteristics

The clinical information of the patients from whom fibroblasts were obtained is detailed in Table 1 [31,32]. All of the mitochondrial disease subjects selected for this study were pediatric patients exhibiting a range of clinical symptoms from mild myopathy to LS to severe neonatal lactic acidosis. All patients carried inherited pathogenic point mutations in mtDNA. One subject (SBG5-FB) was diagnosed with severe neonatal lactic acidosis at the time of disease onset and had the most impaired respiration of all fibroblast lines used in this study. We cultured six fibroblast cell lines (five LS: SBG1-FB (*MT-ATP6-T8993G*), SBG2-FB (*MT-ATP6-T8993G*), SBG3-FB (*MT-ATP6-T9185C*), SBG4-FB (*MT-ND3-T10158C*), SBG5-FB (*MT-ND5-T12706C*) one CTL: BJ-FB) at passage eight, to minimize variability in results. Cells from three biological replicates from each patient line and respective control line were passaged in culture, and samples were frozen and examined at a later time point for genetic analysis.

### 2.2. High Heteroplasmy Was Detected in Disease Lines Affecting ATP Synthase and Low Heteroplasmy Was Detected in Disease Lines Affecting NADH Dehydrogenase

Genomic DNA from different patient and healthy control samples were extracted and mtDNA purified and sequenced (see methods section for details). The percentage of mutant DNA was estimated using high-throughput next-generation sequencing for the whole exome, based on approaches developed in our previous study [33] and detailed in the methods section. The sequencing results yielded a range of total reads to be analyzed between 64 to 302 in the different cell samples. This large sample size allows confidence regarding the percentages measured. The results indicate high heteroplasmy levels between 96% for SBG1-FB (*T8993G*), 91% for SBG2-FB (*T8993G*), and 98% for SBG3-FB (*T9185C*) cell lines containing mutations in *MTATP6* gene impacting complex V. These results were consistent with other published results on *T8993G* and *T9185C* mutations present in high abundance (>90%) that result in MILS [19,34]. Estimation of heteroplasmy for pathogenic mutations in mitochondrial encoded *MTND3* and *MTND5* genes of complex I indicated low levels between 30% for SBG4-FB (*T10158C*) and 39% for SBG5-FB (*T12706C*). The low heteroplasmy level for pathogenic mutation in the *MTND5* gene was consistent with other results in the literature [35]. Since all samples were cultured at passage eight, we observed a lower-than-expected level of heteroplasmy for *T10158C* mutation in this study (Table 2).

### 2.3. Levels of Defective Enzyme Activities of the ETC Correlated with the Percentage of Pathogenic mtDNA

ETC assays measure electron transport through individual components of the respiratory chain [36]. Since the mutations mainly impacted the activity of Complex I and V, we conducted two main assays, one rotenone sensitive and the other rotenone insensitive, to provide measurements of the proximal portion of complex I and a measure of the function of the entire complex [37]. Additional assays included complex III [38] and complex V activity (described in the methods section) in these cell lines. The activity of complex I was estimated in solubilized mitochondria by measuring rotenone sensitive mitochondrial NADH dehydrogenase cytochrome c-reductase (NCR), which was dependent on complexes I-III, and determined the electron transport from donor NADH through complex I, ubiquinone (Q), and complex III where cytochrome c is the electron acceptor. Complex I activity is the rate-limiting step in this assay. The second assay (NFR) is NADH ferricyanide reductase, which measures the reduction of ferricyanide as an artificial electron acceptor, and measures the function of the NADH dehydrogenase portion of complex I.

The results (summarized in Table 3) for cell lines with pathogenic mtDNA variants affecting ATP synthase indicated a statistically significant (*p* < 0.05) increase in NFR activities for SBG1-FB (*T8993G*) by 53%, and SBG3-FB (*T9185C*) by 58% when compared with the control BJ-FB cell line. NFR activity showed a 36% higher trend for the SBG2-FB (*T8993G*) cell line compared with the control BJ-FB cell line. These results suggested a greater capacity for NADH oxidation to NAD+ in these lines. Results for NCR showed increasing trends of 28% for SBG1-FB (*T8993G*) and 31% for SBG3-FB (*T9185C*) lines compared to the control BJ-FB cell line. The increases in NFR and NCR activities suggested a potential compensating effect due to downstream defects in ATP synthase activity. Interestingly, the NCR activity showed a 13% decrease, although not significantly different in the SBG2-FB line carrying (*T8993G*) mutation. The activities of antimycin A-sensitive decylubiquinone-cytochrome-c reductase (complex III) remained similar in SBG2-FB (*T8993G*) and SBG3-FB (*T9185C*) lines compared to the control cell line indicating that complex III activity was not impacted. However, in the SBG1-FB line carrying (*T8993G*) mutation, results showed a significant reduction by ~200% in complex III activity, compared to the control BJ-FB cell line. The activity of oligomycin-sensitive complex V activity showed decreasing trends in cell lines affecting ATP synthase function. Given the low heteroplasmy percentage for pathogenic mtDNA mutations affecting NADH dehydrogenase, results (summarized in Table 3) for cell lines SBG4-FB (*T10158C*) and SBG5-FB (*T12706C*) showed decreasing trends for NFR activities; while NCR, complex III, and oligomycin-sensitive complex V activities remained similar to the control cell line. All the data were normalized to citrate synthase activity, which was used as an indicator of mitochondrial mass in each line.

### 2.4. Mitochondrial Respiration Was Disrupted in Diseased Cell Lines with Variable Spare Respiratory Reserve Capacity

**Figure 1:**
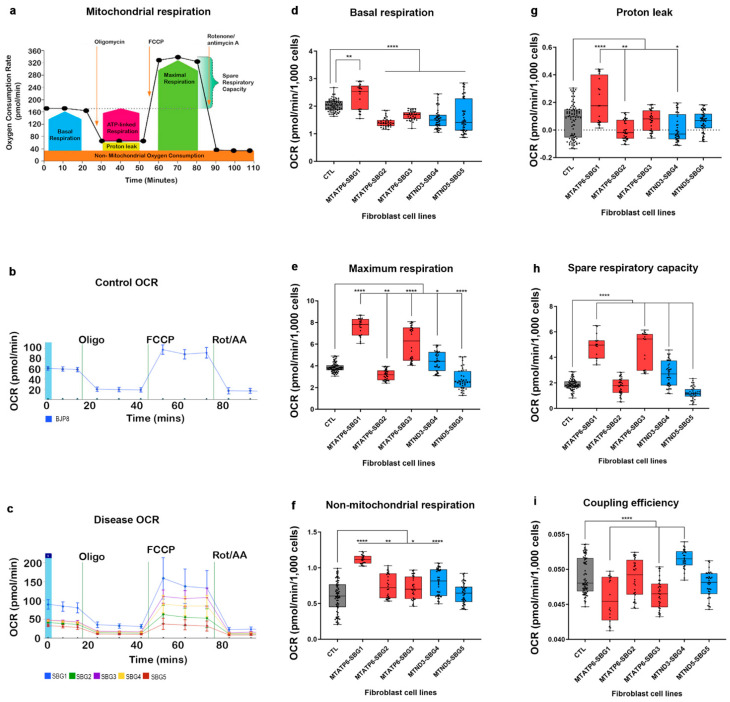
Mitochondrial respiratory profile of CTL BJ-FB and five LS fibroblast cell lines. (**a**) Scheme of expected oxygen consumption rate (OCR) under basal conditions, (**b**) representative OCR profile of BJ-FB, (**c**) representative OCR profile of diseased SBG1-FB (*MT-ATP6-T8993G*), SBG2-FB (*MT-ATP6-T8993G*), SBG3-FB (*MT-ATP6-T9185C*), SBG4-FB (*MT-ND3-T10158C*) and SBG5-FB (*MT-ND5-T12706C*) cell lines showing (**d**) basal respiration, (**e**) maximal respiration (**f**) non- mitochondrial respiration after Rot/AA injection proton leak, (**g**) proton leak (**h**) spare respiratory capacity, (**i**) coupling efficiency. All parameters are in pmol/min/1000 cells. Data are mean +/− SD. Experiments were repeated at least three times on different days under the same conditions. * *p* < 0.05 ** *p* < 0.01 **** *p* < 0.0001. Comparative analyses for all diseased (SBG1-5) FBs were conducted with the healthy control BJ-FB line.

It was hypothesized that an increase in mutation burden disrupts electron transfer (measured as oxygen consumption), leading to altered ETC activity and abnormal mitochondrial bioenergetics. As shown in Figure 1a–c, oxygen consumption rate (OCR) using a Seahorse XFe96 flux analyzer was measured. Analysis was conducted in all cell lines (*n* = 3–5) at passage eight, in conjunction with quantitative measurements of heteroplasmy levels and ETC activity. The oxidative phosphorylation properties included basal respiration, leak, maximal respiration, and non-mitochondrial respiration after sequential injections of ATP synthase inhibitor oligomycin, the uncoupler carbonyl cyanide-4-(trifluoromethoxy) phenylhydrazone FCCP, complex I inhibitor Rotenone, and complex III inhibitor Antimycin A into the wells.

Results showed (Figure 1d) a significant increase (17%; *p* < 0.0001) in basal respiration in the SBG1-FB (*T8993G*) cell line, because disruption of ATP synthase function causes the protons to leak rapidly (Figure 1g) by (195 %; *p* < 0.0001) to maintain the circuit; thus signaling the cells to accelerate the demand for ATP-linked basal respiration. However, proton leak was not increased in SBG2-FB (*T8993G*), SBG3-FB (*T9185C*) cell lines, which correlated with significantly (*p* < 0.0001) decreased basal respiration (Figure 1d,g) when compared to the control line. A similar analysis was performed on the other two cell lines SBG4-FB (*T10158C*) and SBG5-FB (*T12706C*), impacting NADH dehydrogenase function. Results indicated a decrease by 23% and 21% in basal respiration (Figure 1d) for SBG4-FB (*T10158C*) and SBG5-FB (*T12706C*), respectively, relative to the control. Proton leak was also reduced (Figure 1g) in the SBG4-FB line compared to the control BJ-FB cell line.

The maximum respiration rate caused by the addition of FCCP (Figure 1e) showed a significant increase by 98% (*p* < 0.0001) in SBG1-FB(*T8993G*) and 57% (*p* < 0.0001) increase in SBG3-FB (*T9185C*) cell line compared to the control BJ-FB line, mimicking the physiological energy demand due to a defective ATP synthase. Therefore, rapid oxidation of substrates could occur to meet this metabolic challenge, thus stimulating the respiratory chain to operate at maximum capacity [39,40]. However, in the SBG2-FB (*T8993G*) line, the observed decrease by 18% (*p* = 0.0084) in maximal respiration (Figure 1e), indicating the cell was unable to meet the metabolic challenge due to inefficient ATP synthase. Similarly, maximum respiration rate was measured on SBG4-FB (*T10158C*) and SBG5-FB (*T12706C*) cell lines impacting NADH dehydrogenase function. Since the *T12706C* mutation disrupts the proton translocation in the transmembrane arm of the complex I subunit [31], we expected that the maximum rate of respiration would be severely impacted. Results indicate a mild increase in maximum respiration by 15% (*p* < 0.05) in SBG4-FB (*T10158C*) cell line; and a severe decrease in maximum respiration by 29% (*p* < 0.0001) in SBG5-FB (*T12706C*) compared to the control BJ-FB cell line (Figure 1e).

An important bioenergetics variable of a cell that can experience variable energy demands is the spare respiratory capacity (SRC), which is the ability of the electron transport and substrate supply to respond to an increase in ATP demand [41]. SRC is measured by the difference between maximal respiration and basal respiration (Figure 1a). Results showed a significant increase in spare respiratory capacity values by 137% (*p* < 0.0001) in SBG1-FB (*T8993G*) and 142% (*p* < 0.0001) increase in SBG3-FB (*T9185C*) cell line, and a 7% (*p* < 0.0001) decrease in SBG2-FB (*T8993G*) compared with the control BJ-FB cell line. Next, SRC values were measured in SBG4-FB (*T10158C*) and SBG5-FB (*T12706C*) cell lines impacting NADH dehydrogenase function. We observed a significant increase by 45% (*p* < 0.0001) in SRC values in SBG4-FB (*T10158C*) cell line and a significant decrease by 35% (*p* < 0.0001) in SRC values in SBG5-FB (*T12706C*) cell line compared with the control BJ-FB line (Figure 1h). This suggests that the SBG5-FB has a more severe defect in complex I activity versus SBG4-FB. These results are in line with other studies that have shown cells with very low SRC values have poor adaptability to stress conditions [41].

Finally, the mitochondrial respiration was inhibited by simultaneously treating cells with rotenone and antimycin A. Non-mitochondrial respiration was measured as the difference between basal respiration and the final values obtained after the treatment, which is typically attributed to the non-ETC oxidases present in the cell [42]. Results showed that non-mitochondrial respiration was significantly elevated (*p* < 0.0001) in all SBG1-FB (*T8993G*), SBG2-FB (*T8993G*), SBG3-FB (*T9185C*) (*p* < 0.05), SBG4-FB (*T10158C*) cell lines compared to the control BJ-FB line (Figure 1f). The non-mitochondrial respiration was, however, similar in the SBG5-FB (*T12706C*) line when compared with the BJ-FB control cell line.

### 2.5. Glycolytic Rate Is Significantly Increased in SBG1, 3, and 5 Fibroblast Diseased Cell Lines

**Figure 2:**
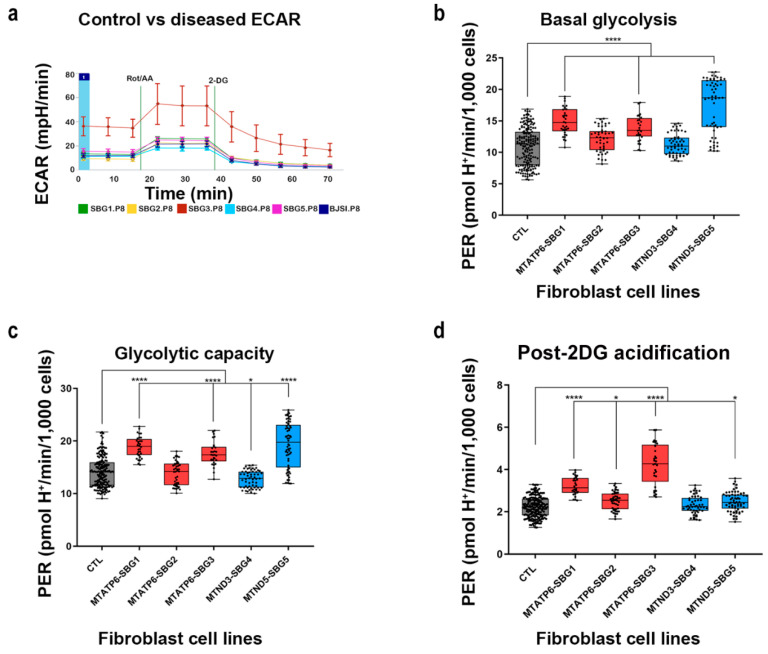
Glycolytic profile of CTL BJ-FB and five LS fibroblast cell lines. (**a**) a real-time profile shows the glycolytic acidification; PER of BJ-FB and diseased SBG1-FB (MT-ATP6-T8993G), SBG2-FB (MT-ATP6-T8993G), SBG3-FB (MT-ATP6-T9185C), SBG4-FB (MT-ND3-T10158C), and SBG5-FB (MT-ND5-T12706C) cell lines showing (**b**) basal glycolysis (**c**) glycolytic capacity after ETC blocking using Rot/AA (**d**) post 2-DG (non-glycolytic acidification). Data are shown in pmol H +/min/1000 cells as mean +/− SD. Experiments were repeated at least three times on different days under the same conditions. * *p* < 0.05 **** *p* < 0.0001. Comparative analyses for all diseased (SBG1-5) FBs were conducted with the healthy control BJ-FB line. PER: proton efflux rate.

**Figure 3:**
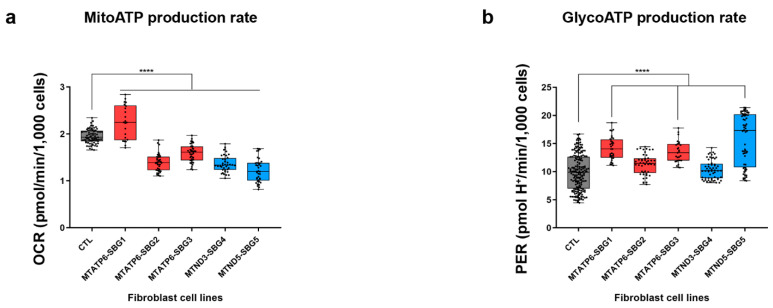
Production of ATP in BJ-FB and five LS fibroblast cell lines. The LS lines are SBG1-FB (*MT-ATP6-T8993G*), SBG2-FB (*MT-ATP6-T8993G*), SBG3-FB (*MT-ATP6-T9185C*), SBG4-FB (*MT-ND3-T10158C*), and SBG5-FB (*MT-ND5-T12706C*), impacting the function of ATP synthase or NADH dehydrogenase. (**a**) Mitochondrial ATP rate and (**b**) Glyco-ATP rate. Data are shown in pmol H +/min/1000 cells as mean +/− SD. Experiments were repeated at least three times on different days under the same conditions. **** *p* < 0.0001. Comparative analyses for all diseased (SBG1-5) FBs were conducted with the healthy control BJ-FB line. OCR: oxygen consumption rate. PER: proton efflux rate.

The mitochondrial bioenergetics assay confirmed that all the patient lines except SBG1-FB (T8993G) showed significant decreases (~35%) in mitochondrial ATP production (Figure 3a) in line with other reports [19,35,43]. Since clinical data from LS patients showed elevated lactate levels [43], a glycolytic rate assay was performed in these lines and compared with the control BJ-FB line. This allowed for the measurement of basal proton efflux rate (PER), a measure of pH change from glycolysis only and not by carbon dioxide and water in the mitochondria via the citric acid cycle and oxidative phosphorylation (see schematics Figure 2a). At the end of the assay, basal glycolysis, glycolytic capacity, and non-glycolytic respiration values were obtained. Basal glycolysis, a measure of basal extracellular proton efflux (PER), was significantly elevated (*p* < 0.0001) by 36% in SBG1-FB (*T8993G*) and 26% in SBG3-FB (*T9185C*) and 70% in SBG5-FB (*T12706C*) line compared to the control BJ-FB line (Figure 2b). Increasing trends were observed in basal glycolysis rates in SBG2-FB (*T8993G*) and SBG4-FB (*T10158C*) lines when compared with the control BJ-FB cell lines.

The addition of mitochondrial inhibitors, rotenone, and antimycin A, forced cells to compensate solely through the glycolytic pathway. Glycolytic capacity, a measure of this compensatory change in cellular metabolism, increased by 34%, 25%, and 37% (*p* < 0.0001) in SBG1-FB (*T8993G*), SBG3-FB (*T9185C*), and SBG5-FB (*T12706C*) lines, respectively, when compared to the control BJ-FB cell line (Figure 2c), further supporting the glycolytic dependence of these cell lines. As observed with the basal glycolysis, glycolytic capacity in the SBG2-FB (*T8993G*) and SBG4-FB (*T10158C*) cell lines were not significantly different from the control BJ-FB cell line. Finally, post-2-DG acidification was recorded after treating the cells with 2-deoxyglucose, to inhibit glycolysis. Post-2-DG acidification, a measure of proton efflux not associated with either mitochondrial respiration or glycolysis, rose by 44% in SBG1-FB (*T8993G*), 12% in SBG2-FB (*T8993G*), and 91% in SBG3-FB (*T9185C*) (*p* < 0.05) cell lines with a defective ATP synthase function (Figure 2d). The post-2-DG acidification was unchanged compared to the control line in SBG4-FB (*T10158C*) while 11% elevated (*p* < 0.05) in SBG5-FB (*T12706C*) mutant fibroblast cell lines.

### 2.6. Mitochondrial ATP Synthesis Rate Is Decreased While Glycolytic ATP Synthesis Rate Is Elevated in Diseased Cell Lines

The defect in the function of ATP synthase or NADH dehydrogenase resulted in a decrease in mitochondrial ATP synthesis rate (Figure 3a) and a subsequent increase in glycolytic ATP synthesis rate (Figure 3b). As expected, we observed significant decreases in mitochondrial ATP synthesis rate (*p* < 0.05) by 29% in SBG2-FB (*T8993G*), 19% in SBG3-FB (*T9185C*), 30% SBG4-FB (*T10158C*), and 37% in SBG5-FB (*T12706C*) lines compared to the control BJ-FB cell lines. Although SBG1-FB (*T8993G*) had a 14% increase in mitochondrial ATP synthesis rate, it was due to compensatory oxygen flux [44]. The glycolytic ATP production rate was increased by 43% in SBG1-FB (*T8993G*), 36% in SBG3-FB (*T9185C*), and 60% in SBG5-FB (*T12706C*) when compared to control BJ-FB cells, supporting the prediction that dysfunctional mitochondrial-derived ATP, results in activation of adaptive pathways of ATP production. The glycolysis ATP rate was similar in SBG2-FB (*T8993G*) and SBG4-FB (*T10158C*) cell lines compared with the control cells (Figure 3b) with less severe defects.

### 2.7. Glycolytic Bioenergetic Health Index (BHI) Is a Sensitive Marker for Predicting Disease Severity in All Cell Lines

**Figure 4:**
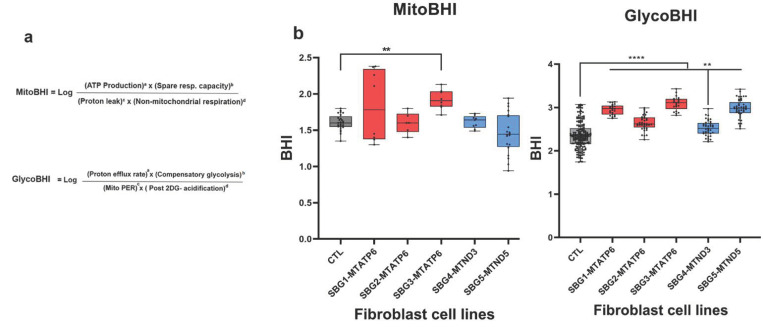
Composite Bioenergetic Health Index (BHI) ratio for BJ-FB and five LS fibroblast cell lines. The LS lines are SBG1-FB (*MT-ATP6-T8993G*), SBG2-FB (*MT-ATP6-T8993G*), SBG3-FB (*MT-ATP6-T9185C*), SBG4-FB (*MT-ND3-T10158C*), and SBG5-FB (*MT-ND5-T12706C*) impacting the function of ATP synthase or NADH dehydrogenase. (**a**) The formula used to calculate the MitoBHI and GlycoBHI (**b**) Mito-BHI values were quantitated based on four bioenergetic parameters: mitoATP production, spare reserve capacity, proton leak and non-mitochondrial respiration. Glyco-BHI values were quantitated based on four glycolytic parameters: Basal glycolysis (Basal PER), compensatory glycolysis, mitochondrial acidification (MitoPER), and post 2-DG acidification. ** *p* < 0.01 **** *p* < 0.0001.

To understand the relationship between disease severity and cellular markers of mitochondrial dysfunction, a single value concept termed “bioenergetics health index or BHI” was recently proposed as a biomarker for measuring overall mitochondrial dysfunction [45,46]. The BHI value was based on mitochondrial bioenergetics parameters and captured positive aspects of bioenergetics function (SRC and ATP-linked respiration) and contrasted these with potentially deleterious aspects (non-mitochondrial oxygen consumption and proton leak) shown in the formula in (Figure 4a) [45]. In this study, the BHI based on mitochondrial parameters were designated as ‘mitoBHI.’ Individual ‘mitoBHI’ values as per the formula in Figure 4a was highest for SBG3-FB (*T9185C*) at 1.92, followed by SBG1-FB (*T8993G*) at 1.83 and 1.61 for both SBG2-FB (*T8993G*) and SBG4-FB (*T10158C*), and SBG5-FB (*T12706C*) had a reduced value of 1.46, while the control BJ-FB cell line was estimated to be at 1.61 (Figure 4b). The ‘mitoBHI’ values in this study were found to be highly variable.

Since the results obtained with the diseased cells also demonstrated a reliance on glycolysis, we introduced a similar concept that we have designated as ‘glycoBHI.’ Similar to the ‘mitoBHI,’ the ‘glycoBHI’ captures positive aspects of glycolysis (Basal PER, compensatory glycolysis) and contrasts these with potentially deleterious non-glycolytic parameters (mitoPER and post 2DG-acidification) (Figure 4a). The first term in the numerator for the glycoBHI is the Basal PER, which refers to the proton efflux rate in live cells by glycolysis only. The second term in the numerator, compensatory glycolysis, is the rate of glycolysis in cells after the addition of mitochondrial inhibitors, which inhibits mitochondrial bioenergetics and drives compensatory changes in the cell to use glycolysis to meet the cells’ energy demands. For the denominator, the mitoPER refers to the contribution of the mitochondria to oxygen consumption and proton production, which is non-glycolytic. The final term in the denominator is the post-2-DG-acidification, which includes other sources of extracellular acidification that are not attributed to glycolysis or mitochondrial TCA activity, as well as any residual glycolysis not fully inhibited by 2-DG. The terms a, b, c, and d in both ‘mitoBHI’ and ‘glycoBHI’ are exponents (linear in log-space), which modify the relative weighting of the respiratory parameters [46]. Individual glycolytic ‘glycoBHI’ values (Figure 4b) were calculated using the four parameters as per the formula in Figure 4a. All ‘glycoBHI’ values for diseased FBs were statistically significant and higher when compared with the control BJ-FB line (Figure 4b). The high ‘glycoBHI’ values corresponded to 3.09, 2.97, and 2.95 for SBG3-FB (*T9185C*), SBG5-FB (*T12706C*), and SBG1-FB (*T8993G*), respectively, which aligns with high basal glycolysis values (Figure 2b). In support of the basal glycolysis and glycolytic capacity data (Figure 2b,c), the ‘glycoBHI’ values were 2.65 and 2.52 for SBG2-FB (*T8993G*) and SBG4-FB (*T10158C*), respectively. Consistent with our findings, the ‘glycoBHI’ values exhibited a 25%, 12%, 31%, 7%, and 26% increase for SBG1-FB, SBG2-FB, SBG3-FB, SBG4-FB, and SBG5-FB, respectively, when compared with the control BJ-FB cell line (Figure 4b).

## 3. Discussion

To better understand and connect the mitochondrial dysfunction contributing to clinical severity in LS, we selected five patient fibroblast lines from young children with mtDNA variants with clinical presentations ranging from mild myopathies to severe LS. The three cell lines contained pathogenic mtDNA with point mutations in the *MT-ATP6* gene [SBG1-FB(*T8993G*), SBG2-FB(*T8993G*), SBG3-FB(*T9185C*)] affecting the function of ATP synthase. The other two cell lines contained pathogenic mtDNA with point mutations in the *MT-ND3* gene [SBG4-FB(*T10158C*)] and the *MT-ND5* gene [SBG5-FB(*T12706C*)], affecting the function of complex I. A commercially available fibroblast cell line (BJ-FB) was used as the control line to minimize variability, and all experiments were designed and analyzed at the same early passage (P8) in this study.

**Figure 5:**
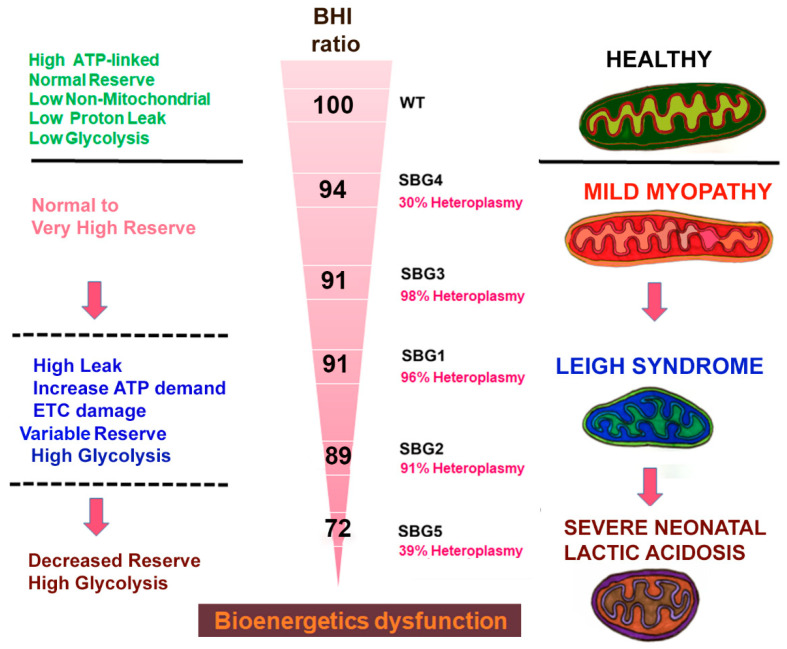
A model for predicting disease severity in LS. Our results indicate that during stress triggered by specific pathogenic mtDNA variants or other factors, cells SBG4-FB (*MT-ND3-T10158C*) with high spare reserve capacity (SRC), low heteroplasmy, and high composite BHI ratio exhibit delayed onset and mild clinical symptoms. However, as SRC and composite BHI ratio decreases, cells SBG5-FB (*MT-ND5-T12706C*) are unable to handle stress and exhibit early-onset and severe clinical symptoms despite low heteroplasmy levels. Whereas cells carrying pathogenic disease mtDNA variants in ATP6 gene SBG1-FB (*MT-ATP6-T8993G*), SBG2-FB (*MT-ATP6- T8993G*), SBG3-FB (*MT-ATP6-T9185C*) exhibit very high heteroplasmy levels, lower composite BHI ratio when compared with control BJ-FB, and can be grouped as ‘intermediate’ in disease severity.

In this study, for the first time, we introduced ‘glycoBHI’ as a more significant and sensitive indicator of the cells (Figure 4b) that have mitochondrial dysfunction. In our study, we noted that the mitoBHI was highly variable, perhaps due to the mtDNA variants, heteroplasmy, or the functional aspects of the specific bioenergetics parameters. Since the disease fibroblasts had adapted and were dependent on both OXPHOS and glycolysis, we also computed one composite BHI ratio (‘mitoBHI’/‘glycoBHI’), based on the averages of the individual replicates associated with the mitoBHI and the glycoBHI for each line. This allowed us to evaluate the overall ability of the cell to meet energy demand due to its diseased state. In this study, we estimated the values to be 0.68 for the control BJ-FB, 0.64 for SBG4-FB, 0.62 for SBG3-FB, 0.62 for SBG1-FB, 0.61 for SBG2 and 0.49 for SBG5-FB. With the value for BJ-FB set at 100 (normal), the composite BHI ratio values were 94 for SBG4-FB (mild), 91 for SBG3-FB (intermediate), 91 for SBG1-FB (intermediate), 89 for SBG2-FB (intermediate) and 72 for SBG5-FB (severe), respectively (Figure 5).

Given the clinical variability and bioenergetic differences among patients with mitochondrial disorders, and LS in particular (as illustrated in Figure 5), an important first step was to comprehensively analyze the range of bioenergetic parameters regulating BHI ratio across the five lines and compare it with the BJ-FB control. By using the XFe96 extracellular flux analyzer, we were able to measure OCR as an indicator of mitochondrial respiration and PER for glycolysis. Mitochondrial and glycolysis specific inhibitors were used to measure different parameters such as basal respiration, ATP turnover, maximal respiration, spare respiratory reserve capacity, proton leak, non-mitochondrial respiration, proton efflux rate, compensatory glycolysis, and ATP production rates in real-time.

In this study, we analyzed two major pathways involved in cellular respiration and ATP production. The first, mitochondrial respiration (OXPHOS), the pathway of ATP generation in the presence of oxygen. The second, basal glycolysis (PER), is the cell’s pathway to synthesize ATP, which is non-mitochondrial. Overall, results demonstrate that SBG2-FB (*T8993G*), SBG3-FB (*T9185C*), SBG4-FB (*T10158C*), and SBG5-FB (*T12706C*) have decreased mitochondrial respiration and ATP production, while SBG1-FB (*T8993G*) cell line was driven by compensatory oxygen flux, most likely due to proton leak across the inner mitochondrial membrane. Although all diseased FBs adapted to the mitochondrial defects with variable increases in the glycolytic pathway, SBG1-FB, SBG3-FB, and SBG5-FB showed significantly higher basal glycolysis (Figure 2b), glycolytic capacity (Figure 2c); and ‘glycoBHI’ (Figure 4b) when compared to the control BJ-FB. SBG2-FB and SBG4-FB showed only a mild increase in basal glycolysis (Figure 2b) and ‘glycoBHI’ (Figure 4b) values when compared with the control BJ-FB. A question then may arise as to why do SBG1-FB, SBG3-FB, and SBG5-FB cells activate the glycolytic pathway? What was the mechanism driving it?

We hypothesized that SRC values were an important contributing factor in the cell’s decision to compensate for the lowered mitochondrial ATP rate and activate the glycolytic pathway. In support of our hypothesis, previous studies reported that a decrease in SRC negatively affects cardiac muscles, making them more vulnerable to bioenergetic exhaustion [47]. Studies on mouse myocytes and iPSC-derived myocytes further support the importance of SRC in cell survival [48]. Other studies also showed that SRC depends on the oxidation of glucose-derived pyruvate [41,48], which is then oxidized into Acetyl-CoA and enters the TCA cycle to produce reduced electron carries, NADH and FAD, which enters the ETC complex to ultimately produce ATP, via the ATP synthase. In line with these findings, results in this study indicated that the LS diseased cell lines demonstrated a decrease in mitochondrial ATP synthesis rate (Figure 3a), prompting a need to activate the glycolysis pathway, perhaps for continued maintenance of high SRC levels (137% and 142%), seen in SBG1-FB (*T8993G*) and SBG3-FB (*T9185C*) cells (Figure 1h). However, SBG5-FB (*T12706C*) cells displayed significantly low (35%) SRC levels (Figure 1h) yet very high (70%) glycolysis rate (Figure 2b). In this scenario, the cells likely have a severe defect in complex I [31] and switch to the glycolysis pathway to satisfy the energy requirement of the cells. Therefore, the low SRC levels perhaps diverted the fate of pyruvate to produce lactate during glycolysis, which supports the clinical outcomes associated with LS [31]. The other cell line with significantly low (7%) SRC levels below the threshold (Figure 1h) was SBG2-FB (*T8993G*). Here the cells triggered a mild increase in basal glycolysis (Figure 2b) to generate pyruvate for minimally maintaining SRC levels. SRC is measured as oxygen consumption in the presence of uncoupler-FCCP and is an indicator of ETC’s capacity to move protons from the mitochondrial matrix into the intermembrane space [49] in the uncoupled state [44]. Therefore, increasing defects in ATP synthase compromised the ETC capacity, leading to a collapse in membrane potential with a concomitant increase in complex I activity indicated by NFR (Table 3).

Lastly, a modest increase (Figure 1h) in SRC levels in SBG4-FB (*T10158C*) cells compared with the control BJ-FB line. SBG4-FB (*T10158C*) cells did not increase basal glycolysis (Figure 2b) for ATP synthesis because of the milder complex I defect or low heteroplasmy levels of 30% (Table 2). Here, we showed for the first time in LS the importance of SRC as a balancing factor in promoting cell adaptability due to functional mitochondrial defects. More importantly, for the first time, we also demonstrate the relevance of glycolysis and the ‘glycoBHI’ as a very significant and sensitive index of mitochondrial defects in the LS diseased lines.

Taking all the cellular dysfunction in LS patient cells into consideration, we have attempted to group the clinical severity presented in Table 1 and Figure 5 into severe, intermediate, and mild categories. In this study, we note that the most severely affected line is SBG5-FB (*T12706C*), based on the comprehensive assessment of bioenergetic parameters (Figure 1, Figure 2, Figure 3 and Figure 4). The SBG5 (*T12706C*) showed a decrease in integrated respiration present in in situ, intact mitochondria (Figure 1d,e) respiring on endogenous substrates. This mitochondrial defect surprisingly was not due to a decrease in complex I enzyme activity measured in solubilized membranes (Table 3). The increase in basal glycolysis confirmed the physiologic impact of the observed decrease in integrated respiration (Figure 2b). The cell respiration assays confirmed that SBG5-FB (*T12706C*) exhibited the lowest composite BHI ratio value of 72 (Figure 5) due to decreased SRC and high glycolysis. Furthermore, the maximal respiration (Figure 1e) and SRC (Figure 1h) were significantly reduced (~35%) when compared to the healthy control BJ-FB, leading to a low ‘mitoBHI’ of 1.46 (Figure 4b). In order to compensate for the loss in mitochondrial ATP production by OXPHOS, the SBG5-FB (*T12706C*) cells adapt and increase glycolysis derived ATP (Figure 3b), leading to a high ‘glycoBHI’ value of 2.97 (Figure 4b). It is thus not surprising that our findings are in agreement with the clinical diagnosis of ‘severe neonatal lactic acidosis’ and resulting in a fatality at 9.5 months within a month of clinical diagnosis [31]. The identification of the specific mechanism of the defect will require further investigation.

Based on our comprehensive assessment of the different bioenergetic parameters, we grouped three cell lines SBG1-FB (*T8993G*), SBG2-FB (*T8993G*), and SBG3-FB (*T9185C*), as ‘intermediate’ with respect to the severity of the disease. Detailed below are the subtle dissimilarities between the three lines to distinguish the bioenergetic differences that contribute to the composite BHI ratios. The first intermediate-to-severe diseased line was SBG2-FB (*T8993G*) with a clinical diagnosis of ‘developmental delay and abnormal gait’ (Figure 5). Although SBG2-FB (*T8993G*) carries the same mutation as SBG1-FB (*T8993G*), the patient exhibited different levels of disease severity reflected in the glycoBHI of 2.64 and a composite BHI ratio value of 89 (Figure 5). Other studies from our laboratory have also shown that an additional uncoupling defect exists in the SBG2-FB cell, which affects the mitochondrial structure and could explain some of the observed bioenergetics differences between SBG1-FB and SBG2-FB [50,51]. We also observed a decrease of 29% in mitochondrial ATP synthesis rate (Figure 3a), the SRC (7%) (Figure 1h), and maximal respiration (18%) (Figure 1e) when compared with the control BJ-FB line. When the SRC is consumed and decreases below the threshold, the mitochondria can no longer respond to energetic demand; thus likely contributing to developmental defects in high-energy tissues such as the brain, heart, and muscle.

The second intermediate diseased line is SBG1-FB (*T8993G*), with a clinical diagnosis as LS (Figure 5) [32], a ‘glycoBHI’ of 2.95, and a composite BHI ratio of 91 (Figure 5). Furthermore, the maximal respiration rate increased significantly by 98% (Figure 1e) and SRC by 137% (Figure 1h) when compared to the control BJ-FB line. However, results also showed an increase in proton leak by 195% when compared with the control BJ-FB (Figure 1g), indicating a ‘pathological level of uncoupling of the respiration state’ [44]. Thus the mitochondria appear to compensate by significantly increasing the basal glycolysis (PER) by 36% (Figure 2b) to preserve the ATP production rate (Figure 3a). However, the increase in glycolytic capacity by 34% (Figure 2c) and the trend to increased glycolytic ATP production rate (Figure 3b) support that glycolytic compensation is likely incomplete. It is also possible that the pyruvate is diverted to produce lactate to preserve glycolytic rates, leading to elevated blood lactate levels as seen in LS patients [2,22].

The third intermediate-to-mild disease variant is SBG3-FB (*T9185C*), in which the patient was diagnosed at age 23 years with ‘mild myopathy’ (Figure 5). The cell respiration assays confirmed that SBG3-FB (*T9185C*) exhibited the highest increase in SRC (142%) (Figure 1h) and increased maximal respiration (57%) (Figure 1e), compared to the control BJ-FB; while the glycolytic ATP was mildly elevated (Figure 3b), accompanied by a glycoBHI of 3.09 and composite BHI ratio of 91 (Figure 5), representing a milder form of LS. The significant increase in post-2DG-acidification (Figure 2d) indicates the possibility of other pathways not attributed to glycolysis or TCA cycle activity being activated to maintain high SRC levels and is worthy of future investigation.

Finally, the mild disease variant is SBG4-FB (*T10158C*) with a clinical diagnosis of ‘epilepsy, dystonic tetraparesis,’ a glycoBHI of 2.52, and a composite BHI ratio of 94 (Figure 5). Furthermore, we observed a slight increase in maximal respiration (15%) (Figure 1e) and SRC (Figure 1h), which indicated that the cells were barely able to meet energy demands by either increasing ATP production within the mitochondria (Figure 3a) or by adapting to the glycolytic pathway. The glycolytic capacity (Figure 2c) and basal glycolysis (PER) (Figure 2b) were only mildly higher when compared with the control line, possibly contributing to a very mild clinical phenotype in the LS patient.

## 4. Materials and Methods

### 4.1. Fibroblast Cell Culture

The clinical information associated with the 5 patient fibroblasts is summarized in Table 1. The first (SBG1) patient was a girl with a clinical presentation of Leigh syndrome, while the second (SBG2) patient was a boy with a clinical presentation of developmental defects. Pathogenic mtDNA mutation *T8993G* was present in the ATP6 gene in both the patient cell lines. The third (SBG3) patient was a girl with a mild clinical myopathy with pathogenic mtDNA mutation *T9185C* was present in the ATP6 gene. The fourth (SBG4) patient was a boy with clinical presentation of epilepsy with pathogenic mtDNA mutation *T10158C* present in mitochondrial ND3 gene, the core subunit of complex I. The fifth (SBG5) patient was a girl with clinical presentation of severe neonatal lactic acidosis with pathogenic mtDNA mutation *T12706C* present in mitochondrial ND5 gene, in the core subunit of complex I. Unaffected healthy control (BJ-FB-ATCC^®^ CRL-2522^TM^) fibroblast cell line was obtained from the American Type Culture Collection (ATCC, Manassas, VA, USA).

Cultures of healthy control and 4 patient-derived diseased fibroblast cell lines were maintained in a fibroblast expansion medium that consisted of minimal essential medium (MEM) (Thermo Fisher Scientific, Waltham, MA, USA) supplemented with 10% fetal bovine serum (FBS) (GE healthcare-HyClone^TM^; Chicago, IL, USA) and 2mM L-glutamine (Thermo Fisher Scientific, Waltham, MA, USA). All cell lines were cultured and maintained at 37 °C in a humidified atmosphere of 5% CO_2_. The culture medium was replenished every 2 days and passaged when cells reached 80% confluence. Fibroblasts were enzymatically passaged in 0.05% Trypsin-EDTA (Thermo Fisher Scientific, Waltham, MA, USA). All experiments were performed with cells at passage 8 for consistency and to minimize experimental variability.

### 4.2. Next-Generation Sequencing for Heteroplasmy Analysis

Frozen cell pellets from different samples containing ~2 × 10^6^ cells were thawed and processed. The QIAamp DNA mini kit (Qiagen, Valencia, CA, USA) manufacturer protocol was followed to extract total DNA, which resulted in elution of 100 μL of distilled water (dH2O) and total DNA from all cells. The 100 μL solution containing the genomic DNA was further treated with 1 μL of RNaseA for 1 h at 37 °C to avoid RNA contamination. The gDNA was quantified using DeNovix UV/Vis Spectrophotometer (DeNovix Inc., Wilmington, DE, USA). A blank of 1.0 μL of dH2O was used to establish a zero, and 1.0 μL of each sample was used to determine the concentration.

The DNA concentration was verified using a Qubit fluorometer (Thermo Fisher Scientific, Waltham, MA, USA). Instead of the standard DNA fragmentation, an enzymatic fragmentation was performed using the KAPA Frag Enzyme from the KAPA HyperPlus Library Preparation Kit (KAPA Biosystems, Wilmington, MA, USA). This alternative was performed to increase yield during the fragmentation step. Fragmented DNA was purified using Ampure beads (Beckman Coulter, Brea, CA, USA). DNA libraries were prepared using the Accel-NGS 2S Plus DNA Library Kit (Swift Biosciences, Ann Arbor, MI, USA). Ten PCR cycles were carried out during the Library Amplification step. The final libraries were analyzed with a 2100 Bioanalyzer to assess library size distribution (Agilent Technologies, Santa Clara, CA, USA). DNA libraries were quantified with the KAPA Library Quantification Kit to ensure accuracy (KAPA Biosystems). Based on the qPCR results, the DNA libraries were compiled in equimolar amounts and sequenced with the HiSeq 2500 using TruSeq v3 reagents according to the 2 × 100 bp protocol (Illumina, San Diego, CA, USA).

Heteroplasmy fractions were extracted from the FASTQ files as previously described T8993G [52]. Briefly, we first filtered out reads that were likely to be nuclear mitochondrial sequences (NuMTs). NuMTs are DNA sequences that are harbored in the nuclear genome but closely match sequences in the mitochondrial genome [53]. Specifically, reads that aligned, using the Burrows–Wheeler Alignment Tool [54], with up to one mismatch to the nuclear reference sequence GRCh38 (having removed the mitochondrial revised Cambridge reference sequence (rCRS) [55], were excluded from downstream analysis. The resulting reads were realigned to rCRS, and read counts of the mutant and wild-type alleles were extracted using SAMtools mpileup [56]. From these counts, the mutant heteroplasmy level was computed as: (mutant allele counts)/(total counts).

### 4.3. Mitochondrial Oxygen Consumption Detection, Glycolysis Function Test, and Bioenergetics Health Index (BHI)

In this study, we evaluated the metabolic state in the patient-derived fibroblasts to further understand the influence of mtDNA mutations on cellular bioenergetics. Changes in oxygen consumption were measured in real-time using an XFe96 extracellular flux analyzer. Seahorse XF96 Cell Mito Stress Test Kit and glycolytic rate assay kit (Agilent, Santa Clara, CA, USA) were used as per the manufacturer’s instructions. Prior to use in XFe96, fibroblasts were detached using mild trypsin and seeded into the plates with a previously optimized number of 20,000 cells per well. All fibroblasts were seeded in 10–12 replicate wells per plate, with the experiment repeated at least 4–5 times.

The cells were supplemented with 180 μL Mito-stress complete Seahorse medium, after which the cells were incubated in a non-CO_2_ incubator at 37 °C for one hour. Respiration was measured using the classic mitochondrial inhibitors, specific for complex I and III subunits, such as Rotenone and Antimycin A (0.5 μM final concentrations each). Maximum respiration was measured by the addition of an uncoupler carbonyl cyanide-4-(trifluoromethoxy) phenylhydrazone- FCCP (0.7 μM final concentration); and Oligomycin (1 μM final concentration) was added to measure proton leak. The readouts were normalized to cell numbers and analyzed using the Seahorse XF96 Wave software (ver 2.6, Agilent, Santa Clara, CA, USA).

Given the presence of point mutations impacting *ATP6, ND5,* and *ND3*, we also analyzed glycolytic function in all fibroblasts. A classical glycolytic rate assay was performed using the XFe96 based on the following procedure: (1) cells were cultured in buffered (5 mM HEPES buffer) Seahorse medium supplemented with glucose and pyruvate; (2) the proton efflux rate (PER) was measured after the addition of saturating amounts of glucose; (3) rotenone and antimycin A were added to inhibit mitochondrial-derived ADP phosphorylation; and (4) 2-DG was added to inhibit glycolysis. The different assay parameters: basal glycolysis, compensatory glycolysis, total proton efflux, and post 2-DG acidification, were normalized to cell number and analyzed using Seahorse XFe96 Wave software.

The mitochondrial-derived bioenergetic health index (mitoBHI), a composite index of mitochondrial quality, was determined using the formula in Figure 4a, where a, b, c, and d exponents modify the relative weight of each respiratory parameter and by default were equivalent to 1 in this experiment. The glycolytic BHI (glycoBHI), an index of glycolytic respiration, was determined using the formula in Figure 4a. The composite BHI index was calculated by taking a ratio of the means of mitoBHI and glycoBHI.

### 4.4. Measurement of Electron Transport Chain Activity

The activity of electron transport chain complexes I, III, and V was measured in frozen-thawed, detergent-solubilized fibroblast cell lines. The overall approach of Hoppel and colleagues was used [57,58]. The assay was optimized for the use of smaller volume incubations (300 µL) with preliminary experiments first using isolated mouse heart mitochondria, followed by the use of H9c2 cardiomyoblast cells. In these initial experiments, the solubilization method and volumes used preserved complex activity compared to the use of larger assay volumes and greater amounts of mitochondrial and cellular protein. The amount of cell protein used was based on initial work that established linear ranges of total enzyme activity vs. protein content. The fibroblast cell lines SBG 1-5 and control non-diseased fibroblasts were maintained as above. They were pelleted by centrifugation [400× *g*] and flash-frozen with liquid nitrogen. They were shipped by overnight express from SI to EJL and immediately placed at −80 °C. On the day of the experiment cells (approximately 2–5 million cells) were thawed on ice, suspended, and centrifuged at 10,000 rcf. The supernatant was removed, and pellets were suspended in 4 °C mannitol (220 mM)-sucrose (70 mM)-MOPS (5 mM) buffer with Na_2_EDTA (2 mM), pH 7.4. Sodium cholate hydrate (5 g/100 mL Milli-Q water) was added, the mixture briefly vortexed, 2 additional volumes of MSM-EDTA were added to decrease the final cholate concentration to 1%. Protein content was measured using the Lowry assay. Citrate synthase activity was measured using 10 µg of cell protein by the oxaloacetate mediated reduction of DTNB at 412 nm. Complex I activity was assessed using NADH:ferricyanide oxidoreductase (NFR) and NADH:cytochrome c oxidoreductase (NCR; rotenone sensitive). NFR was measured using the artificial electron acceptor potassium ferricyanide using 50 µg of cell protein at 340 nm by following the decrease in NADH content. NCR was measured by following the rotenone-sensitive reduction of cytochrome c using the increase in absorbance at 550 nm using 100 μg of cell protein. NCR measures the activities of complex I and III with complex I the rate-limiting step. Complex III activity (decylubiquinol: cytochrome c oxidoreductase, antimycin A sensitive) was measured using 50 µg of cell protein at 550 nm by following the increase in the content of reduced cytochrome c. Complex V activity (oligomycin sensitive) was measured using 45 µg of cell protein using a commercially available kit (Cayman Chemical, Ann Arbor, MI, USA) according to the manufacturer’s instructions.

### 4.5. Statistical Analysis

In order to ensure scientific rigor and reproducibility, for the bioenergetics analysis, an ANOVA design accounting for 4–5 biological and 10–12 technical replicated from control (BJ-FB) and diseased (SBG1-FB, SBG2-FB, SBG3-FB, SBG4-FB, SBG5-FB) that were nested within groups was used to identify any differences with respect to control BJ-FBs. Post-hoc Tukey HSD tests were used to identify differences among specific groups. Data were presented as the mean ± standard deviation (SD) and were analyzed using the GraphPad Prism 8 software (GraphPad Software, San Diego, CA, USA). A *p* < 0.05 was considered significant.

## 5. Conclusions

Based on the overall analysis of the five diseased patient-specific fibroblasts, the ‘glycoBHI’ emerged as a sensitive indicator of mitochondrial defects as the cells had switched ‘on’ the glycolytic pathway. As shown in Figure 4b, glycoBHI was significantly increased in all the cell lines compared to control BJ-FB and was indeed sensitive to mitochondrial dysfunction.

We also computed the ‘composite BHI ratio’ oxphos/glycolysis because the cell lines were utilizing both oxphos (although highly defective) and glycolysis pathways to maintain the energy requirements in the individual cell line. As noted in Figure 5, the composite BHI ratio (100 was deemed healthy), while the decreased composite BHI ratio (72 was unhealthy), and indicative of a very disease. Two important parameters associated with the composite BHI ratio were basal glycolysis (PER), which was a measure of mitochondrial defect, and SRC, which was an indicator of the cell’s capacity to adapt to the defect.

Another important consideration is pathogenic mtDNA variants and heteroplasmy levels associated with the specific mtDNA variant. The current study indicates that in addition to the bioenergetic parameters, there must be consideration of the specific pathogenic mtDNA variant, rather than solely relying on the percentage heteroplasmy. In line with previous studies [21,32,59], we also measured a high heteroplasmy for pathogenic mtDNA MT-ATP6 variants SBG1-FB: 96%; SBG2-FB: 91%; SBG3-FB: 98%, that is associated with LS, and impacting ATP synthase function. As other studies have reported [32,59], the clinical phenotype for SBG1-FB-*MT-ATP6-T8993G* was classic ‘LS’, while for SBG2-FB *MT-ATP6-T8993G* was ‘developmental delay, abnormal gait’; and for SBG3-FB-*MT-ATP6-T9185C* a mild myopathy and late disease onset. Despite the high heteroplasmy and identical mutation, the composite BHI ratio was able to distinguish the different MT-ATP6 variants with regard to the disease severity.

Previous studies [29,60] showed that the pathogenic *MT-ND3-T10158C* and *MT-ND5-T12706C* mtDNA variants have also been observed in patients exhibiting LS phenotype. In the current study, heteroplasmy was measured to be 30% in the SBG4-FB-*MT-ND3-T10158C*. The subtle increase in SRC (Figure 1h), lowered basal respiration (Figure 1d) and basal glycolysis (Figure 2b) was in line with a milder metabolic defect manifested as epilepsy and muscle weakness for the patient from whom SBG4-FB was derived. Heteroplasmy was measured at 39% in SBG5-FB-MT-ND5-T12706C, associated with the most severe clinical phenotype. Previous studies reported varying heteroplasmy levels (from 30–70%) for this mtDNA variant with severe clinical phenotypes associated with LS, which indicates that even a low mutation burden could contribute to disease severity [61,62,63]. The present study demonstrates that SBG5-FB exhibited low SRC (Figure 1h); low mitochondrial ATP production (Figure 3a), very high glycolytic ATP production (Figure 3b); and high basal glycolysis (Figure 2b), which was in line with the observed clinical phenotype of neonatal lactic acidosis. In the current study, we showed that low heteroplasmy in SBG4- FB-*MT-ND3-T10158C* contributed to a milder clinical phenotype, while in SBG5-FB-*MT-ND5-T12706C* contributed to a very severe phenotype in the context of LS. However, the composite BHI ratio emerged as a comprehensive biomarker based on the value of 94 for SBG4-FB- *MT-ND3-T10158C* and 72 for SBG5-FB-*MT-ND5-T12706C* and correlated with the severity of the clinical phenotype (Figure 5). Overall, these results suggest that as long as the precise mechanism of LS has not been elucidated, a multi-pronged approach that takes into consideration the specific pathogenic mtDNA variant, along with a composite BHI ratio, can aid in better diagnosis and understanding the factors influencing disease severity and rapid fatality in LS.

Another possibility suggested by our work beyond mitochondrial disorders is incorporating the composite BHI ratio as a measure of overall mitochondrial health and fitness in different cell types utilizing both glycolytic and oxidative phosphorylation pathways to maintain energy production. Specifically, in adult metabolic disorders such as diabetes, cancer, or obesity, our findings here suggest that drugs that target mitochondrial SRC [64,65,66,67] could hold promise as therapeutic potential and might warrant further study for their ability to alter ATP levels and restore the BHI ratio to physiological levels.

## Figures and Tables

**Table 1:** Clinical information of five patient fibroblast cell lines with pathogenic mtDNA mutations [31,32] and CTL control cell line.

Sample Name	Mutation	Gene	Clinical Information	Age at Diagnosis	Sex
BJ-FB	None	-		-	M
SBG1-FB	*T8993G*	*MTATP6*	Leigh syndrome	3 years	F
SBG2-FB	*T8993G*	*MTATP6*	Developmental delay, abnormal gait	4 years	M
SBG3-FB	*T9185C*	*MTATP6*	Myopathy	23 years	F
SBG4-FB	*T10158C*	*MTND3*	Epilepsy, dystonic tetraparesis	9 years	M
SBG5-FB	*T12706C*	*MTND5*	Severe neonatal lactic acidosis	8 months	F

**Table 2:** Quantification of heteroplasmy by next-generation sequencing. The extracted mtDNA was sequenced using whole-exome sequencing methods. The sequencing results were compiled and analyzed as detailed in the methods section. This allows us to compute heteroplasmic variants based on the sequencing reads. Results demonstrate the presence of pathogenic mtDNA burden in all LS fibroblast samples.

Sample Name	Mutation	Total Reads Analyzed	Normal	Variant	Percentage of Mutation
SBG1-FB	*T8993G*	136	6(T)	130(G)	96%
SBG2-FB	*T8993G*	68	6(T)	62(G)	91%
SBG3-FB	*T9185C*	302	6(T)	296(C)	98%
SBG4-FB	*T10158C*	64	45(T)	19 (C)	30%
SBG5-FB	*T12706C*	146	89(T)	57(C)	39%

**Table 3:** Electron transport chain activity of CTL BJ-FB and five LS fibroblast cell lines. The electron transport chain activities for NFR (proximal part of CI), NCR (distal part of CI), CIII, CV of mitochondria isolated from control and diseased cells are normalized to citrate synthase activity measured in the same samples. Activities are expressed in nmol/mg protein/min/CS. Results are mean +/− S.D, *n* = 3–5 for all individual disease lines, and *n* = 9 for the BJ-FB control cell line. NCR, NADH:cytochrome c oxidoreductase; NFR, NADH:ferricyanide oxidoreductase; CS, citrate synthase; CIII, cytochrome bc1; CV, ATP synthase. * *p* < 0.05 *** *p* < 0.001 **** *p* < 0.0001 vs. control.

Sample Name	CS	NFR	NCR	CIII	CV
CTL-BJ-FB	42.22 ± 13.1	16.08 ± 5.5	0.051 ± 0.04	0.67 ± 0.17	0.080 ± 0.12
SBG1-FB	31.00 ± 5.4	34.02 ± 3.8 ***	0.071 ± 0.04	0.29 ± 0.16 *	0.063 ± 0.039
SBG2-FB	38.00 ± 7.4	25.48 ± 8.0	0.045 ± 0.06	0.60 ± 0.20	0.030 ± 0.018
SBG3-FB	26.75 ± 5.6	38.60 ± 5.6 ****	0.103 ± 0.08	0.66 ± 0.24	0.086 ± 0.060
SBG4-FB	44.50 ± 9.2	15.83 ± 0.92	0.053 ± 0.0097	0.75 ± 0.23	0.038 ± 0.013
SBG5-FB	41.40 ± 8.26	13.48 ± 1.91	0.063 ± 0.014	0.50 ± 0.16	0.042 ± 0.0033

## Data Availability

No dataset has been deposited in a repository, and the data from the studied patient fibroblasts are not publicly available, in agreement with privacy law and our institutional policies.

## References

[1] McFarland R., Taylor R.W., Turnbull D.M. (2010). A neurological perspective on mitochondrial disease. Lancet Neurol..

[2] Bakare A.B., Lesnefsky E.J., Iyer S. (2021). Leigh Syndrome: A Tale of Two Genomes. Front. Physiol..

[3] Schaefer A.M., Taylor R.W., Turnbull D.M., Chinnery P.F. (2004). The epidemiology of mitochondrial disorders--past, present and future. Biochim. Biophys. Acta.

[4] Skladal D., Halliday J., Thorburn D.R. (2003). Minimum birth prevalence of mitochondrial respiratory chain disorders in children. Brain.

[5] Wallace D.C., Chalkia D. (2013). Mitochondrial DNA genetics and the heteroplasmy conundrum in evolution and disease. Cold Spring Harb. Perspect. Biol..

[6] Coller H.A., Bodyak N.D., Khrapko K. (2002). Frequent intracellular clonal expansions of somatic mtDNA mutations: Significance and mechanisms. Ann. N. Y. Acad. Sci..

[7] Nekhaeva E., Bodyak N.D., Kraytsberg Y., McGrath S.B., Van Orsouw N.J., Pluzhnikov A., Wei J.Y., Vijg J., Khrapko K. (2002). Clonally expanded mtDNA point mutations are abundant in individual cells of human tissues. Proc. Natl. Acad. Sci. USA.

[8] Nekhaeva E., Kraytsberg Y., Khrapko K. (2002). mtLOH (mitochondrial loss of heteroplasmy), aging, and ‘surrogate self’. Mech. Ageing Dev..

[9] Mitchell P. (1961). Coupling of phosphorylation to electron and hydrogen transfer by a chemi-osmotic type of mechanism. Nature.

[10] Nicholls D.G., Ferguson S.J. (2013). Bioenergetics 4.

[11] Murphy M.P. (2009). How mitochondria produce reactive oxygen species. Biochem. J..

[12] Iyer S., Bergquist K., Young K., Gnaiger E., Rao R.R., Bennett J.P. (2012). Mitochondrial gene therapy improves respiration, biogenesis, and transcription in G11778A Leber’s hereditary optic neuropathy and T8993G Leigh’s syndrome cells. Hum. Gene Ther..

[13] Jain I.H., Zazzeron L., Goli R., Alexa K., Schatzman-Bone S., Dhillon H., Goldberger O., Peng J., Shalem O., Sanjana N.E. (2016). Hypoxia as a therapy for mitochondrial disease. Science.

[14] Baertling F., Rodenburg R.J., Schaper J., Smeitink J.A., Koopman W.J., Mayatepek E., Morava E., Distelmaier F. (2014). A guide to diagnosis and treatment of Leigh syndrome. J. Neurol. Neurosurg. Psychiatry.

[15] Holt I.J., Harding A.E., Petty R.K., Morgan-Hughes J.A. (1990). A new mitochondrial disease associated with mitochondrial DNA heteroplasmy. Am. J. Hum. Genet..

[16] Leigh D. (1951). Subacute necrotizing encephalomyelopathy in an infant. J. Neurol. Neurosurg. Psychiatry.

[17] Loeffen J.L., Smeitink J.A., Trijbels J.M., Janssen A.J., Triepels R.H., Sengers R.C., van den Heuvel L.P. (2000). Isolated complex I deficiency in children: Clinical, biochemical and genetic aspects. Hum. Mutat..

[18] Rahman J., Noronha A., Thiele I., Rahman S. (2017). Leigh map: A novel computational diagnostic resource for mitochondrial disease. Ann. Neurol..

[19] Schubert Baldo M., Vilarinho L. (2020). Molecular basis of Leigh syndrome: A current look. Orphanet J. Rare Dis..

[20] Shoffner J.M., Fernhoff P.M., Krawiecki N.S., Caplan D.B., Holt P.J., Koontz D.A., Takei Y., Newman N.J., Ortiz R.G., Polak M. (1992). Subacute necrotizing encephalopathy: Oxidative phosphorylation defects and the ATPase 6 point mutation. Neurology.

[21] Tatuch Y., Christodoulou J., Feigenbaum A., Clarke J.T., Wherret J., Smith C., Rudd N., Petrova-Benedict R., Robinson B.H. (1992). Heteroplasmic mtDNA mutation (T----G) at 8993 can cause Leigh disease when the percentage of abnormal mtDNA is high. Am. J. Hum. Genet..

[22] Lake N.J., Compton A.G., Rahman S., Thorburn D.R. (2016). Leigh syndrome: One disorder, more than 75 monogenic causes. Ann. Neurol..

[23] Debray F.G., Lambert M., Lortie A., Vanasse M., Mitchell G.A. (2007). Long-term outcome of Leigh syndrome caused by the NARP-T8993C mtDNA mutation. Am. J. Med. Genet. A.

[24] Moslemi A.R., Darin N., Tulinius M., Oldfors A., Holme E. (2005). Two new mutations in the MTATP6 gene associated with Leigh syndrome. Neuropediatrics.

[25] Uziel G., Moroni I., Lamantea E., Fratta G.M., Ciceri E., Carrara F., Zeviani M. (1997). Mitochondrial disease associated with the T8993G mutation of the mitochondrial ATPase 6 gene: A clinical, biochemical, and molecular study in six families. J. Neurol. Neurosurg. Psychiatry.

[26] Rahman J., Rahman S. (2018). Mitochondrial medicine in the omics era. Lancet.

[27] Rahman S. (2020). Mitochondrial disease in children. J. Intern. Med..

[28] Piekutowska-Abramczuk D., Rutyna R., Czyzyk E., Jurkiewicz E., Iwanicka-Pronicka K., Rokicki D., Stachowicz S., Strzemecka J., Guz W., Gawronski M. (2018). Leigh syndrome in individuals bearing m.9185T>C MTATP6 variant. Is hyperventilation a factor which starts its development?. Metab. Brain Dis..

[29] Kirby D.M., Salemi R., Sugiana C., Ohtake A., Parry L., Bell K.M., Kirk E.P., Boneh A., Taylor R.W., Dahl H.H. (2004). NDUFS6 mutations are a novel cause of lethal neonatal mitochondrial complex I deficiency. J. Clin. Investig..

[30] Fassone E., Rahman S. (2012). Complex I deficiency: Clinical features, biochemistry and molecular genetics. J. Med. Genet..

[31] Ni Y., Hagras M.A., Konstantopoulou V., Mayr J.A., Stuchebrukhov A.A., Meierhofer D. (2019). Mutations in NDUFS1 Cause Metabolic Reprogramming and Disruption of the Electron Transfer. Cells.

[32] Stendel C., Neuhofer C., Floride E., Yuqing S., Ganetzky R.D., Park J., Freisinger P., Kornblum C., Kleinle S., Schols L. (2020). Delineating MT-ATP6-associated disease: From isolated neuropathy to early onset neurodegeneration. Neurol. Genet..

[33] Grace H.E., Galdun P., Lesnefsky E.J., West F.D., Iyer S. (2019). mRNA Reprogramming of T8993G Leigh’s Syndrome Fibroblast Cells to Create Induced Pluripotent Stem Cell Models for Mitochondrial Disorders. Stem Cells Dev..

[34] Childs A.M., Hutchin T., Pysden K., Highet L., Bamford J., Livingston J., Crow Y.J. (2007). Variable phenotype including Leigh syndrome with a 9185T>C mutation in the MTATP6 gene. Neuropediatrics.

[35] Kirby D.M., Boneh A., Chow C.W., Ohtake A., Ryan M.T., Thyagarajan D., Thorburn D.R. (2003). Low mutant load of mitochondrial DNA G13513A mutation can cause Leigh’s disease. Ann. Neurol..

[36] Chance B., Williams G.R. (1955). Respiratory enzymes in oxidative phosphorylation. IV. The respiratory chain. J. Biol. Chem..

[37] Hoppel C.L., Kerr D.S., Dahms B., Roessmann U. (1987). Deficiency of the reduced nicotinamide adenine dinucleotide dehydrogenase component of complex I of mitochondrial electron transport. Fatal infantile lactic acidosis and hypermetabolism with skeletal-cardiac myopathy and encephalopathy. J. Clin. Investig..

[38] Hatefi Y. (1978). Preparation and properties of dihydroubiquinone: Cytochrome c oxidoreductase (complex III). Methods Enzymol..

[39] Smith R.L., Soeters M.R., Wust R.C.I., Houtkooper R.H. (2018). Metabolic Flexibility as an Adaptation to Energy Resources and Requirements in Health and Disease. Endocr. Rev..

[40] Zorov D.B., Juhaszova M., Sollott S.J. (2014). Mitochondrial reactive oxygen species (ROS) and ROS-induced ROS release. Physiol. Rev..

[41] Marchetti P., Fovez Q., Germain N., Khamari R., Kluza J. (2020). Mitochondrial spare respiratory capacity: Mechanisms, regulation, and significance in non-transformed and cancer cells. FASEB J..

[42] Hill B.G., Benavides G.A., Lancaster J.R., Ballinger S., Dell’Italia L., Jianhua Z., Darley-Usmar V.M. (2012). Integration of cellular bioenergetics with mitochondrial quality control and autophagy. Biol. Chem..

[43] Crimi M., Papadimitriou A., Galbiati S., Palamidou P., Fortunato F., Bordoni A., Papandreou U., Papadimitriou D., Hadjigeorgiou G.M., Drogari E. (2004). A new mitochondrial DNA mutation in ND3 gene causing severe Leigh syndrome with early lethality. Pediatr. Res..

[44] Gnaiger E., MitoEAGLE T.G. (2020). Mitochondrial physiology. Bioenerg. Commun..

[45] Chacko B.K., Kramer P.A., Ravi S., Benavides G.A., Mitchell T., Dranka B.P., Ferrick D., Singal A.K., Ballinger S.W., Bailey S.M. (2014). The Bioenergetic Health Index: A new concept in mitochondrial translational research. Clin. Sci..

[46] Chacko B.K., Zhi D., Darley-Usmar V.M., Mitchell T. (2016). The Bioenergetic Health Index is a sensitive measure of oxidative stress in human monocytes. Redox Biol..

[47] Gong G., Liu J., Liang P., Guo T., Hu Q., Ochiai K., Hou M., Ye Y., Wu X., Mansoor A. (2003). Oxidative capacity in failing hearts. Am. J. Physiol. Heart Circ. Physiol..

[48] Pfleger J., He M., Abdellatif M. (2015). Mitochondrial complex II is a source of the reserve respiratory capacity that is regulated by metabolic sensors and promotes cell survival. Cell Death Dis..

[49] Nicholls D.G., Darley-Usmar V.M., Wu M., Jensen P.B., Rogers G.W., Ferrick D.A. (2010). Bioenergetic profile experiment using C2C12 myoblast cells. J. Vis. Exp..

[50] Bakare A.B., Rao R.R., Iyer S. (2021). Cell-Permeable Succinate Increases Mitochondrial Membrane Potential and Glycolysis in Leigh Syndrome Patient Fibroblasts. Cells.

[51] Bakare A.B., Daniel J., Stabach J., Rojas A., Bell A., Henry B., Iyer S. (2021). Quantifying Mitochondrial Dynamics in Patient Fibroblasts with Multiple Developmental Defects and Mitochondrial Disorders. Int. J. Mol. Sci..

[52] Grandhi S., Bosworth C., Maddox W., Sensiba C., Akhavanfard S., Ni Y., LaFramboise T. (2017). Heteroplasmic shifts in tumor mitochondrial genomes reveal tissue-specific signals of relaxed and positive selection. Hum. Mol. Genet..

[53] Hazkani-Covo E., Zeller R.M., Martin W. (2010). Molecular poltergeists: Mitochondrial DNA copies (numts) in sequenced nuclear genomes. PLoS Genet..

[54] Li H., Durbin R. (2009). Fast and accurate short read alignment with Burrows-Wheeler transform. Bioinformatics.

[55] Andrews R.M., Kubacka I., Chinnery P.F., Lightowlers R.N., Turnbull D.M., Howell N. (1999). Reanalysis and revision of the Cambridge reference sequence for human mitochondrial DNA. Nat. Genet..

[56] Li H., Handsaker B., Wysoker A., Fennell T., Ruan J., Homer N., Marth G., Abecasis G., Durbin R., Genome Project Data Processing S. (2009). The Sequence Alignment/Map format and SAMtools. Bioinformatics.

[57] Krahenbuhl S., Chang M., Brass E.P., Hoppel C.L. (1991). Decreased activities of ubiquinol:ferricytochrome c oxidoreductase (complex III) and ferrocytochrome c:oxygen oxidoreductase (complex IV) in liver mitochondria from rats with hydroxycobalamin[c-lactam]-induced methylmalonic aciduria. J. Biol. Chem..

[58] Lesnefsky E.J., Tandler B., Ye J., Slabe T.J., Turkaly J., Hoppel C.L. (1997). Myocardial ischemia decreases oxidative phosphorylation through cytochrome oxidase in subsarcolemmal mitochondria. Am. J. Physiol.

[59] Ganetzky R.D., Stendel C., McCormick E.M., Zolkipli-Cunningham Z., Goldstein A.C., Klopstock T., Falk M.J. (2019). MT-ATP6 mitochondrial disease variants: Phenotypic and biochemical features analysis in 218 published cases and cohort of 14 new cases. Hum. Mutat..

[60] McFarland R., Kirby D.M., Fowler K.J., Ohtake A., Ryan M.T., Amor D.J., Fletcher J.M., Dixon J.W., Collins F.A., Turnbull D.M. (2004). De novo mutations in the mitochondrial ND3 gene as a cause of infantile mitochondrial encephalopathy and complex I deficiency. Ann. Neurol..

[61] Lebon S., Chol M., Benit P., Mugnier C., Chretien D., Giurgea I., Kern I., Girardin E., Hertz-Pannier L., de Lonlay P. (2003). Recurrent de novo mitochondrial DNA mutations in respiratory chain deficiency. J. Med. Genet..

[62] Taylor R.W., Morris A.A., Hutchinson M., Turnbull D.M. (2002). Leigh disease associated with a novel mitochondrial DNA ND5 mutation. Eur. J. Hum. Genet..

[63] Zhadanov S.I., Grechanina E.Y., Grechanina Y.B., Gusar V.A., Fedoseeva N.P., Lebon S., Munnich A., Schurr T.G. (2007). Fatal manifestation of a de novo ND5 mutation: Insights into the pathogenetic mechanisms of mtDNA ND5 gene defects. Mitochondrion.

[64] Corazao-Rozas P., Guerreschi P., Andre F., Gabert P.E., Lancel S., Dekiouk S., Fontaine D., Tardivel M., Savina A., Quesnel B. (2016). Mitochondrial oxidative phosphorylation controls cancer cell’s life and death decisions upon exposure to MAPK inhibitors. Oncotarget.

[65] Roy Chowdhury S.K., Smith D.R., Saleh A., Schapansky J., Marquez A., Gomes S., Akude E., Morrow D., Calcutt N.A., Fernyhough P. (2012). Impaired adenosine monophosphate-activated protein kinase signalling in dorsal root ganglia neurons is linked to mitochondrial dysfunction and peripheral neuropathy in diabetes. Brain.

[66] Yamamoto H., Morino K., Mengistu L., Ishibashi T., Kiriyama K., Ikami T., Maegawa H. (2016). Amla Enhances Mitochondrial Spare Respiratory Capacity by Increasing Mitochondrial Biogenesis and Antioxidant Systems in a Murine Skeletal Muscle Cell Line. Oxid Med. Cell. Longev..

[67] Zhang G., Frederick D.T., Wu L., Wei Z., Krepler C., Srinivasan S., Chae Y.C., Xu X., Choi H., Dimwamwa E. (2016). Targeting mitochondrial biogenesis to overcome drug resistance to MAPK inhibitors. J. Clin. Investig..

